# A novel multicolor immunostaining method using ethynyl deoxyuridine for analysis of in situ immunoproliferative response

**DOI:** 10.1007/s00418-015-1329-z

**Published:** 2015-05-15

**Authors:** Yusuke Kitazawa, Hisashi Ueta, Thomas Hünig, Yasushi Sawanobori, Kenjiro Matsuno

**Affiliations:** Department of Anatomy (Macro), Dokkyo Medical University, 880 Kitakobayashi, Mibu, Tochigi 321-0293 Japan; Institute for Virology and Immunobiology, University of Würzburg, Würzburg, Germany

**Keywords:** EdU, Multicolor immunofluorescence staining, *Rat*, Immunohistology, Flow cytometry, Immunoproliferative response, T-cell dendritic cell cluster

## Abstract

Immune responses are generally accompanied by antigen presentation and proliferation and differentiation of antigen-specific lymphocytes (immunoproliferation), but analysis of these events in situ on tissue sections is very difficult. We have developed a new method of simultaneous multicolor immunofluorescence staining for immunohistology and flow cytometry using a thymidine analogue, 5-ethynyl-2′-deoxyuridine (EdU). Because of the small size of azide dye using click chemistry and elimination of DNA denaturation steps, EdU staining allowed for immunofluorescence staining of at least four colors including two different markers on a single-cell surface, which is impossible with the standard 5-bromo-2′-deoxyuridine method. By using two *rat* models, successfully detected parameters were the cluster of differentiation antigens including phenotypic and functional markers of various immune cells, histocompatibility complex antigens, and even some nuclear transcription factors. Proliferating cells could be further sorted and used for RT-PCR analysis. This method thus enables functional in situ time-kinetic analysis of immunoproliferative responses in a distinct domain of the lymphoid organs, which are quantitatively confirmed by flow cytometry.

**Electronic supplementary material:**

The online version of this article (doi:10.1007/s00418-015-1329-z) contains supplementary material, which is available to authorized users.

## Introduction

Immune responses induce activation and clonal expansion of antigen-specific lymphocytes with active DNA synthesis for cell division. Dendritic cells (DCs) undergo a crucial interaction with lymphocytes as professional antigen-presenting cells in the distinct domain of the secondary lymphoid organs. DCs form a cluster with antigen-specific lymphocytes and induce immunoproliferation, i.e., their differentiation and proliferation within the cluster (Matsuno et al. 1989; Saiki et al. 2001; Ueta et al. 2008). Therefore, in situ examination of phenotype and functional molecules of cycling cells and cellular interactions with DCs or stromal cells during the immune response should provide crucial information for understanding immunity in health and diseases.

We have long studied immunoproliferative responses in situ by using a multicolor immunoenzyme staining analysis (Matsuno et al. 1989, 2010; Saiki et al. 2001; Ueta et al. 2008) for a thymidine analogue, 5-bromo-2′-deoxyuridine (BrdU), and other parameters. However, because enzyme-developed color dyes tend to interfere with the following immunostaining step, only two-color analysis prior to BrdU staining could be performed at best and detection of two different markers on a single-cell surface was very difficult. Immunofluorescence staining has an advantage, because fluorescent dyes do not interfere with each other and four-color staining is possible by a standard protocol. However, BrdU immunofluorescence staining is also problematic, because it requires DNA denaturation steps for exposing antigen epitopes by strong acid or heating, resulting in decreased intensity of other fluorescent dyes. Therefore, a new method other than BrdU staining has been needed for a long time.

Although flow cytometry (FCM) enables quantitative analysis of multiple parameters of a proper cell subset, it requires single-cell suspensions and therefore cannot delineate the in situ localization or cellular interaction. In contrast, although immunohistology is the most practical method for in situ analysis, quantitative analysis of the immune response in the tissue sections is time consuming and not easy. Accordingly, parallel analysis of both immunohistology and FCM using one sample should provide good information about the immune response.

Recently, a thymidine analogue, EdU (5-ethynyl-2′-deoxyuridine), was described as a replacement for BrdU to directly measure de novo DNA synthesis of S-phase cycling cells using click chemistry (Salic and Mitchison 2008). Click chemistry is a method of covalently coupling an azide with an alkyne. Detection of EdU relies on the copper (I)-catalyzed click reaction with an azide-modified fluorescent dye to form a stable triazole ring. Because of the small size of the azide dye, no harsh denaturation steps are needed to gain access to the DNA (Salic and Mitchison 2008). Previous studies reported that EdU could be used for immunohistology (Salic and Mitchison 2008) or FCM (Diermeier-Daucher et al. 2009) as a thymidine analogue. Accordingly, EdU staining holds the potential to be applied in multicolor immunofluorescence including proliferating cells and double-membrane staining of a single cell, which is impossible with the standard BrdU method. In neuroscience research, a few publications report using triple immunohistogical staining for two neuronal peptide antigens and EdU (Guo et al. 2009). Because the tissues are prefixed with paraformaldehyde (PFA) and stable intracellular peptide antigens are targets of immunostaining, this method is not applicable for double-membrane staining of the cluster of differentiation (CD) antigens, which are mostly labile and easily denatured or masked by aldehyde fixatives.

In the present study, by applying our original multicolor immunoenzyme (Saiki et al. 2001; Ueta et al. 2008) and immunofluorescence (Sawanobori et al. 2014) staining methods, we have tried to develop a new method of simultaneous multicolor immunofluorescence staining using EdU for up to four colors for immunohistology and up to three colors for FCM. Two models for assessing the in vivo proliferative response of immune cells were examined. The first is the administration of a CD28 superagonist (CD28SA), reported to preferentially expand forkhead box P3 (Foxp3) gene—expressing naturally occurring regulatory T-cells (nTregs) (Beyersdorf et al. 2005). The second is the one-way graft-versus-host reaction (GvHR) by the transferring of parental congeneic T-cells to F_1_ hybrid *rats* (Matsuno et al. 2010). Here we show that lymphocyte markers, histocompatibility complex antigens, cell adhesion molecules, and even nuclear transcription factors in addition to EdU can be detected simultaneously by this method.

## Materials and methods

### Animals

Inbred male Lewis (RT1A^l^B^l^) and PvG/c (RT1A^c^B^c^) *rats* were purchased by SLC Co. (Shizuoka, Japan). Congeneic PVG/c-RT7^b^/OlaHsd (RT1A^c^B^c^RT7^b^) *rats* and (PvG/c × Lewis) F_1_ hybrid *rats* were bred and maintained in the Laboratory Animal Center for Research (Dokkyo Medical University). All animals were reared under specific pathogen-free conditions and used at 8–10 weeks of age. Animal handling and care were approved by the Dokkyo Medical University Animal Experiments Committee and were in accordance with the Dokkyo University’s Regulations for Animal Experiments and with Japanese Governmental Law (No. 105). No studies involving human participants are reported here.

### Antibodies and reagents

Monoclonal antibodies (mAbs) and labeled secondary antibodies (Abs) used for immunohistology and FCM (FACSCalibur, BD Biosciences, Franklin Lakes, NJ, USA) analyses are listed in Table 1. Some mAbs were purified from culture supernatants and coupled to fluorescein isothiocyanate, PerCP/Cy5.5 (Innova Bioscience Ltd, Cambridge, UK), Alexa Fluor^®^ 350 (Alexa-350), 488, 594, 647, or 680 (Molecular Probes, Eugene, OR, USA) in house. To detect EdU, the Click-iT^®^ EdU Alexa-488, -594, or -647 Flow kit for FCM or imaging was used (Click-iT kit, Life Technologies Corporation, Carlsbad, CA, USA).

**Table 1:** Antibodies used in this study

Primary Ab			
Antigen	Isotype	Clone	Conjugate^Source^
TCRαβ	Mouse IgG_1_	R73	Unconjugated^h^, Alexa-647^k #^, PerCP-Cy5.5^l #^
CD4	Mouse IgG_2a_	OX38	Unconjugated^a^
CD8β	Mouse IgG_1_	341	Unconjugated^b^, Alexa-647^b^
CD11c	Mouse IgG_2a_	8A2	Unconjugated^a^
CD25	Mouse IgG_1_	OX39	Unconjugated^h^, fluorescein isothiocyanate (FITC)^b^
CD103 (αE integrin)	Mouse IgG_1_	OX62	Unconjugated^h^, Alexa-594^#^
CD205	Mouse IgG_1_	HD83	Unconjugated^b^, Alexa-594 ^#^
Donor lymphocyte CD45 (RT7^b^)	Mouse IgG_1_	HIS41	Unconjugated^j^, Alexa-488^#^, Alexa-647^#^
Host MHCII (RT1B^l^)	Mouse IgG_1_	OX3	Unconjugated^h^, Alexa-647^#^
Helios	Hamster IgG	22F6	PerCP/Cy5.5^b^
Foxp3	Rat IgG_2a_	FJK-16s	Biotin^d^, Alexa-647^d^
BrdU	Rat IgG_2a_	BU1/75	Unconjugated^a^, Alexa-647^#^
Type IV collagen	Rabbit IgG	Polyclonal	Unconjugated^i^

Secondary Ab and streptavidin		
Product	Conjugate	Source
Goat Ig anti-mouse IgG	Alkaline phosphatase	*g*
Donkey Ig anti-rat IgG	Alkaline phosphatase	*e*
Goat F(ab′)_2_ anti-rabbit IgG	Horseradish peroxidase	*c*
Goat Ig anti-mouse IgG	Alexa-350, Alexa-594, Alexa-680	*f*
Donkey Ig anti-rabbit IgG	Aminomethylcoumarin (AMCA)	*e*
Streptavidin	Alexa-488	*f*

^#^Own conjugation

^a^AbD serotec

^b^Biolegend

^c^Cappel

^d^Ebioscience

^e^Jackson immunoresearch

^f^Life Technologies Corporation

^g^Sigma

^h^ECACC

^i^Generously provided by Dr. Y. Sado

^j^Generously provided by Dr. F. Kroese

^k^Alexa: Alexa Flour^®^

^l^The tandem conjugate in Peridinin Chlorophyll Protein Complex and Cyanine 5.5

### Experimental design

For the first experiment, Lewis *rats* received intravenous injection of a CD28 superagonist mAb (CD28SA, clone JJ316: 0, 0.25, 0.5, 1 mg/300 g body weight), and the spleens were collected 3 days later. In the second experiment, one-way systemic GvHR was induced by intravenous injection of T-cells of congeneic PVG/c-RT7^b^*rats* into (PvG/c × Lewis) F_1_ hybrid *rats*, and the spleens were collected 1 and 2 days after injection. For a source of donor T-cells, thoracic duct lymphocytes (5.0 × 10^7^ cells/*rat*) were used after thoracic duct cannulation, as reported (Zhou et al. 2008). In both experiments, recipient *rats* received an intravenous injection of a mixture of equivalent moles of BrdU (6 mg/200 g body weight, Sigma-Aldrich Japan, Tokyo) and EdU (5 mg/200 g body weight, Life Technologies Corporation) in phosphate-buffered saline (PBS) 1 h before killing. To avoid masking or loss of labile CD antigens by aldehyde fixatives, fresh cryosections without prefixation were employed. General anesthesia during animal procedures was provided using isoflurane (Mylan Inc., Tokyo, Japan) supplied by an isoflurane vaporizer (SN-487-OT; Shinano Manufacturing, Tokyo, Japan).

### Splenic lymphocyte isolation

The harvested spleens were injected with Collagenase D (1 mg/mL, Roche Diagnostics GmbH, Mannheim, Germany) and DNase I (400 U/mL, Roche Diagnostics GmbH) in 3 mL Hank’s buffered salt solution (HBSS) containing 5 % fetal calf serum, 1.2 mM CaCl_2_·2H_2_O, and 0.8 mM MgSO_4_·7H_2_O and were digested under gentle stirring for 30 min at 37 °C in a CO_2_ incubator (MCO-18AIC; Sanyo, Osaka, Japan). The collagenase digestion was stopped by adding 0.5 M EDTA solution and five volumes of cold PBS. The isolated splenocytes were filtered through a 200-μm nylon mesh and washed twice in PBS with 0.2 % bovine serum albumin (PBS-BSA) by centrifugation (himac CF16RX; Hitachi Ltd, Tokyo, Japan) at 280×*g* for 10 min at 4 °C. The splenic lymphocyte fraction was isolated in an OptiPrep discontinuous density gradient (15 and 11.5 %, Axis-Shield, Oslo, Norway) by centrifugation at 600×*g* for 24 min at room temperature (RT). With this approach, the upper layer cells of the 15 % OptiPrep were mainly lymphocytes; interface cells between 15 and 11.5 % OptiPrep were macrophages, and DCs. The lymphocyte fractions were washed once by centrifugation at 440×*g* for 10 min at 4 °C and used for FCM.

### Flow cytometric analysis

Splenic lymphocytes at 10^6^ cells/100 μL PBS-BSA were incubated for 30 min at 4 °C with an optimal concentration of purified mouse mAbs to anti-rat CD antigens diluted and washed three times with PBS-BSA by centrifugation at 350×*g* for 5 min. The cells were incubated with PerCP/Cy5.5-conjugated anti-mouse IgG secondary antibody (Biolegend, San Diego, CA, USA) for 30 min at 4 °C in PBS-BSA with 1 % normal rat serum and rinsed three times with PBS-BSA. Cells then were incubated for 1 h at 4 °C with normal mouse IgG (20 µg/mL) in PBS-BSA for blocking additional mouse antibody binding. The next step was incubation with a purified second mAb directly conjugated with Alexa-647 for 30 min at 4 °C, followed by a wash. The EdU staining was performed at the final step. The cells were permeabilized with a permeabilization buffer set (00-5523-00, eBioscience San Diego, CA, USA) overnight (O/N) at 4 °C for intracellular staining and washed. EdU was visualized using the Click-iT kit for FCM according to the manufacturer’s instructions. Cells were analyzed by FCM (FACSCalibur) with CellQuest Pro software (BD Biosciences).

In case of Foxp3 staining, the second mAb was omitted, and the cells were first permeabilized in the same manner as for EdU staining. Then, the cells were incubated with Alexa-647-conjugated anti-mouse/rat Foxp3 mAb (FJK-16s, eBioscience) in permeabilization buffer for 30 min at 4 °C and washed at least three times with the same buffer. The EdU staining was performed without the permeabilization step.

In the first experiment, TCRαβ^+^CD25^high^EdU^+^ or EdU^−^ cells were further isolated by FACSAria (BD Biosciences) sorting. Then Foxp3 message was examined in both cell groups by reverse transcription polymerase chain reaction (RT-PCR, Model TP600, Takara Bio, Inc., Shiga, Japan) at 30 cycles (for 10 s at 98 °C, 30 s at 60 °C, and 60 s at 72 °C). Primers were as follows: Foxp3, forward primer, 5′-CGG GAG AGT TTC TCA AGC AC-3′; reverse primer, 3′-GGA GCT CTT GTC CAC TGA GG-5′; GAPDH (glyceraldehyde-3-phosphate dehydrogenase: Internal control), forward primer, 5′-AGA CAG CCG CAT CTT CTT GT-3′; and reverse primer, 3′-CTT GCC GTG GGT AGA GTC AT-5′.

### Cryosectioning and pretreatment

We applied the processing method for the immunoenzyme (Saiki et al. 2001; Ueta et al. 2008) and immunofluorescence (Sawanobori et al. 2014) staining methods in our laboratory to the EdU immunofluorescence staining. Fresh cryosections were cut using a LEICA CM1850 (Leica Microsystems, Ontario, Canada) and were processed as described in the “Recommended protocols.”

### Multicolor fluorescence immunohistology using EdU

For both experiments, three- to four-color immunofluorescent staining was performed as precisely described in supplemental online materials for either phenotype (TCRαβ, etc.) of proliferating cells (EdU), nuclear transcription factor (Foxp3), tissue frameworks (type IV collagen), and DCs (CD103, etc.) in the spleen cryosections. Multicolor images were captured using an Axioskop2 Plus fluorescent microscope (Carl Zeiss, Jena, Germany) with an AxioCam MRm camera and AxioVision software (Carl Zeiss). Filters used were Filter Set 49 for Alexa-350, 17 for Alexa-488, 32 for Alexa-647 or -680 (Carl Zeiss), and XF407 for Alexa-594 (Omega Optical, Brattleboro, VT, USA), respectively. This filter combination had negligible crossing over of emitted lights between filters. We assigned pseudocolors to each channel to make merged images more comprehensible by maximizing contrast using AxioVision software (Carl Zeiss).

### Multicolor enzyme immunohistology using BrdU

For the second experiment, the spleen cryosections were triple enzyme-immunostained for donor lymphocytes (RT7^b^ congeneic marker, *blue*), type IV collagen (*brown*), and proliferating cells (BrdU, *red*) as previously described (Saiki et al. 2001; Ueta et al. 2008).

### Correlation of EdU and BrdU in cell proliferation analysis

To confirm that EdU-positive (EdU^+^) cells and BrdU^+^ cells were the same proliferating cell population, triple immunofluorescent staining for type IV collagen, EdU, and BrdU was performed. After the blocking solution, sections were incubated for 1 h ~O/N at RT with a rabbit anti-mouse type IV collagen Ab and washed. Sections were incubated with aminomethylcoumarin (AMCA)-conjugated anti-rabbit IgG for 1 h and washed. Then, EdU was stained using the Click-iT^®^ kit. For DNA denaturation, sections were treated for 10 min at 89 °C by a Microwave processor (MI-77, Azumaya, Tokyo, Japan) with a Retrivagen kit (BD Biosciences) and cooled to RT. After being washed and blocked, sections were incubated with Alexa-647-conjugated anti-BrdU mAb for 1 h at RT. Sections were mounted with coverslips and were examined under a fluorescence microscope.

## Results

### EdU staining correlates well with BrdU staining in immunohistology

The Click-iT^®^ kit resulted in intense and clear EdU staining with a low signal-to-noise ratio for all fluorochromes tested: Alexa-488, -594, and -647. The protocol from the manufacturer was easy and reproducible and applicable not only for FCM but also for immunohistology.

The immunostaining of the spleen in the first experiment showed a massive proliferative response in the white pulp at 3 days after CD28SA stimulation (Supplementary Fig. 1). Double immunofluorescent staining for EdU and BrdU showed superimposition of EdU^+^ nuclei on almost all BrdU^+^ nuclei (Supplementary Fig. 1). To note, in some cells, the intensities of both stainings were different where BrdU^high^ cells are EdU^low^ or vise versa, which was also shown previously (Salic and Mitchison 2008).

### Parallel analysis of FCM and immunohistology

**Fig. 1:**
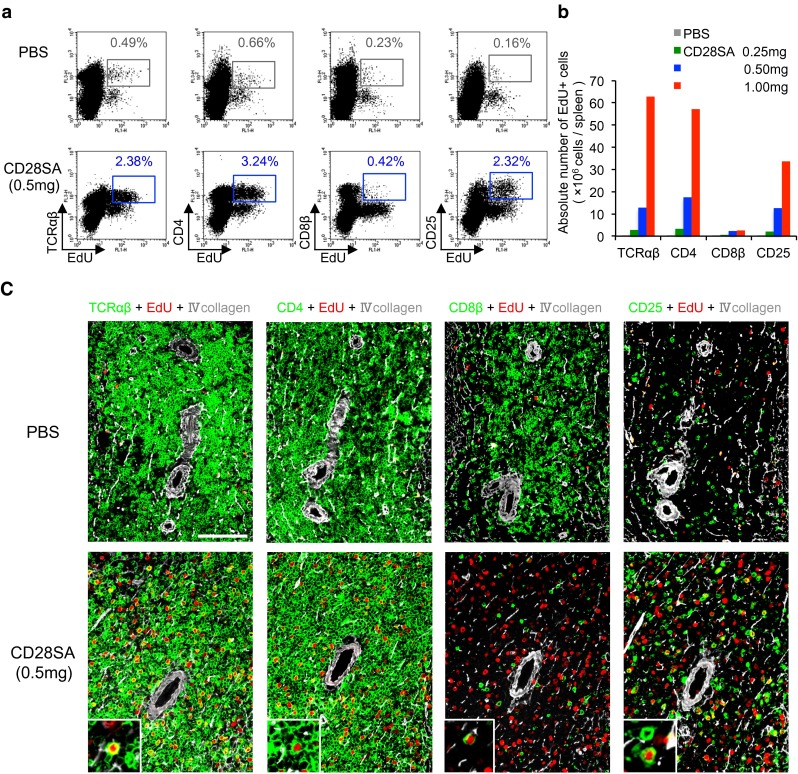
Assessment of proliferating lymphocytes in the spleen activated by CD28SA. **a** FCM analysis of the splenic lymphocytes for lymphocyte markers and EdU at day 3 after CD28SA injection (0.5 mg/*rat*). *Note* increase in TCRαβ^+^, CD4^+^, or CD25^+^ EdU^+^ cells but not CD8β^+^EdU^+^ cells compared to control. **b** Dose response of splenic lymphocytes to CD28SA. Absolute number of EdU^+^ proliferating cells with different lymphocyte markers/spleen, showing 1.0 mg group induces the highest response. **c** Triple immunofluorescent staining for lymphocyte markers (indirect staining with Alexa-680-conjugated anti-mouse IgG, *green*), EdU (Alexa-594-conjugated azide, *red*) and type IV collagen (indirect staining with AMCA-conjugated anti-rabbit IgG, *white*). Day 3 after CD28SA injection (0.5 mg/*rat*). Pseudocolors were assigned using AxioVision software. *Scale bar* 100 µm. The splenic PALS area with the central arteries is depicted by type IV collagen. Proliferating cells with different markers are shown as *green* cells with *red* nuclei (*inset* of *lower panel*)

When the proliferative response of TCRαβ^+^, CD4^+^, CD8β^+^, and CD25^+^ cells was examined by FCM of the spleen cells in the same *rats*, the absolute numbers of EdU^+^TCRαβ^+^, EdU^+^CD4^+^, EdU^+^CD8β^+^, and EdU^+^CD25^+^ cells increased in a dose-dependent fashion relative to CD28SA (Fig. 1a, b). In contrast, EdU^+^CD8β^+^ cells were much fewer in number than other EdU^+^ cells (Fig. 1b).

Multicolor fluorescence immunohistology of *rats* receiving 0.5 mg of CD28SA showed that many TCRαβ^+^, CD4^+^, and CD25^+^ cells but only a few CD8β^+^ cells had EdU^+^ nuclei in the T-cell area of the splenic white pulp, i.e., the periarterial lymphocyte sheath (PALS) (Fig. 1c). Therefore, EdU staining enables the parallel examination of the proliferative response of activated cells both quantitatively by FCM and qualitatively by immunohistology of tissue sections.

### Simultaneous detection of a transcription factor and cycling S-phase cells

**Fig. 2:**
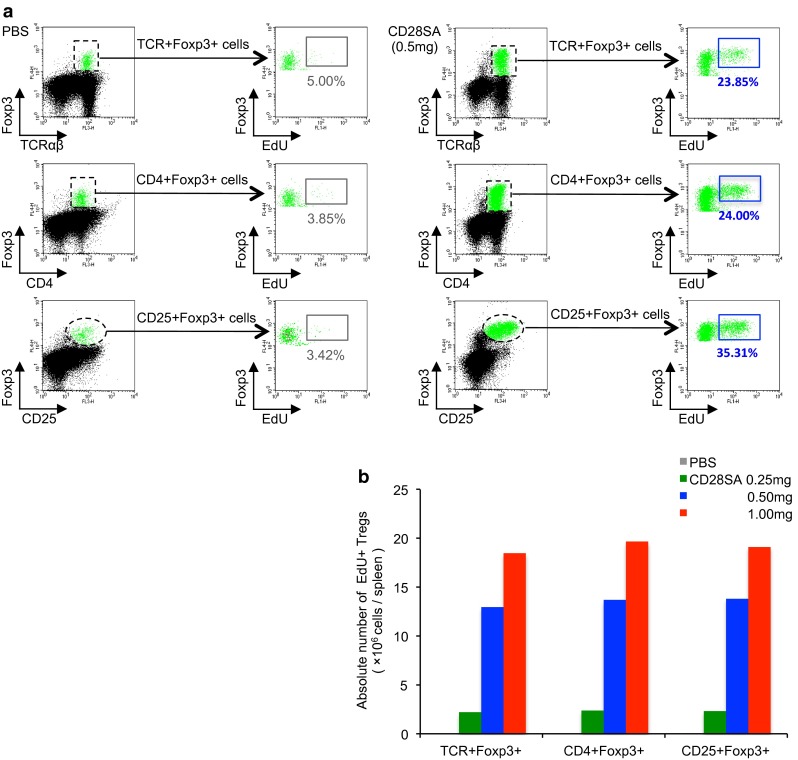
FCM for proliferating Tregs induced by CD28SA. **a** Three-color FCM analysis of the splenic lymphocytes for lymphocyte markers, Treg transcription factor (Foxp3), and EdU at day 3 after CD28SA injection (0.5 mg/*rat*). **b** Dose response of Foxp3^+^Tregs with different markers to CD28SA. Absolute number of EdU^+^ proliferating Tregs with different lymphocyte markers/spleen, showing 0.5–1.0 mg induces an intense response

**Fig. 3:**
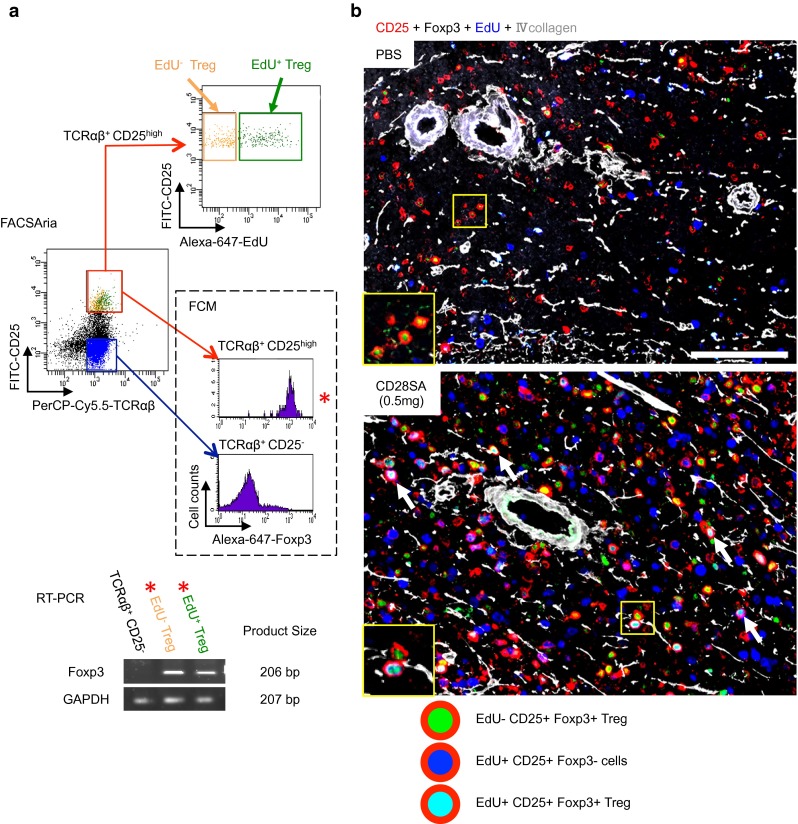
FACSAria sorting and immunohistological analysis for proliferating Tregs. **a** RT-PCR analysis of TCRαβ^+^CD25^high^Foxp3^+^ EdU^+^ or EdU^−^ Tregs isolated by FACSAria sorting. **b** Four-color immunofluorescent staining for CD25 (indirect staining with Alexa-680-conjugated anti-mouse IgG, *red*), Foxp3 (Biotin-labeled mAb plus Alexa-488-conjugated streptavidin, *green*), EdU (Alexa-594-conjugated azide, *blue*), and type IV collagen (indirect staining with AMCA-conjugated anti-rabbit IgG, *white*). Pseudocolors were assigned using AxioVision software. Splenic PALS area at day 3 after CD28SA injection (0.5 mg/*rat*). Proliferating CD25^+^Foxp3^+^ Tregs are depicted as *red* cells with light *blue* nuclei (*inset* and *white arrows*) in the CD28SA spleen but few in the control spleen. *Scale bar* 100 μm

We next examined the nuclear transcription factors of Tregs, i.e., Foxp3 (Kitazawa et al. 2009) and Helios (Gottschalk et al. 2012), with the expectation that expression would increase with CD28SA stimulation. At day 3 after 0.5 mg CD28SA stimulation, the FCM analysis of the spleen cells showed a fivefold to tenfold increase in TCRαβ^+^, CD4^+^, or CD25^+^EdU^+^ cells expressing Foxp3 compared to PBS-injected control (Fig. 2a). The absolute number of proliferating Tregs increased in a dose-dependent fashion with CD28SA (Fig. 2b). In addition, some EdU^+^Foxp3^+^CD25^+^Tregs also expressed Helios (not shown). Furthermore, by using this sample, TCRαβ^+^CD25^high^EdU^+^ or EdU^–^ Tregs could be isolated by FACSAria sorting, and Foxp3 message could be detected in both cell groups by RT-PCR (Fig. 3a).

Multicolor fluorescence immunohistology of the same spleen showed that many CD25^high^ cells with Foxp3^+^EdU^+^ nuclei were more frequently observed in the PALS than in the control (Fig. 3b). The results were in agreement with those of the FCM study.

We thus could directly demonstrate proliferation and expression of some transcription factors of Tregs by FCM and immunohistology. Foxp3^+^EdU^+^ proliferating Tregs could be further isolated and used for RT-PCR analysis.

### Simultaneous detection of two different surface markers of cycling S-phase cells

**Fig. 4:**
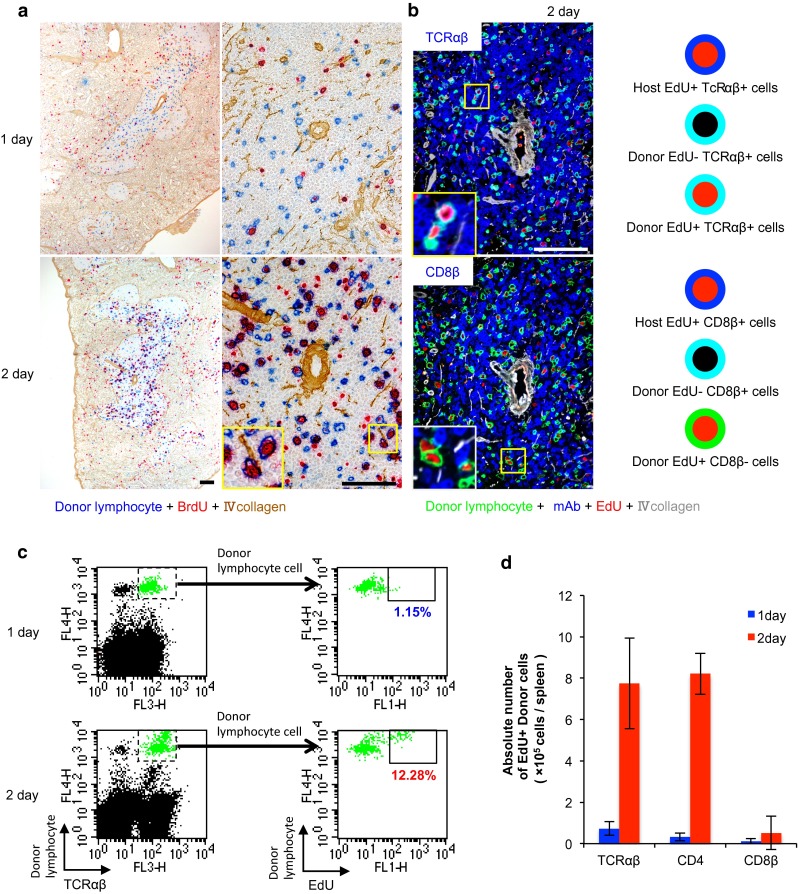
Migration and proliferation of donor lymphocytes in GvHR. **a** Three-color immunoenzyme staining of the spleen after GvHR induction for donor lymphocytes (RT7^b+^, *blue*), type IV collagen (*brown*), and BrdU (*red*). *Note* migration of donor lymphocytes into the PALS on day 1 and their proliferation on day 2 (*inset*). *Scale bar* 100 μm. **b** Four-color immunofluorescence staining of the spleen for TCRαβ or CD8β (Alexa-647-conjugated mAb, *blue*), donor lymphocytes (indirect staining with Alexa-488-conjugated anti-mouse IgG, *green*), type IV collagen (indirect staining with AMCA-conjugated anti-rabbit IgG, *white*), and EdU (Alexa-594-conjugated azide, *red*). Pseudocolors were assigned using AxioVision software. Merged images are explained schematically in the *right side* of the panels. *Note* proliferation of TCRαβ^+^ (*light blue* cell with *red* nucleus, *inset*) but not CD8β^+^ donor cells (no *light blue* cell with *red* nucleus, *inset*). *Scale bar* 100 μm. **c** FCM for donor lymphocytes, TCRαβ, and EdU of the spleen, showing increase in proliferating TCRαβ^+^ donor T-cells on day 2. **d** Absolute number of EdU^+^ proliferating donor lymphocytes with different lymphocyte markers/spleen, showing TCRαβ^+^CD4^+^ donor T-cells actively proliferate on day 2

We used a GvHR model to examine whether EdU can be used or not for analysis of proliferating cells in the immune response. First, three-color enzyme immunostaining was performed to confirm our previously published findings (Matsuno et al. 2010; Zhou et al. 2008). The injected donor cells were detected by staining a congeneic CD45 marker (RT7^b^) by His41 mAb (Kampinga et al. 1990), type IV collagen, and BrdU. One day after injection, RT7^b+^ donor lymphocytes were detected in the host splenic PALS, as confirmed by type IV collagen staining, and a few of them were BrdU^+^ (Fig. 4a). On day 2, many donor lymphocytes became BrdU^+^ (Fig. 4a). This finding represents proliferation of activated donor lymphocytes undergoing GvHR.

Second, four-color immunofluorescence staining of the same *rat* spleens was performed. As expected, many EdU^+^RT7^b+^ donor lymphocytes were TCRαβ^+^ (Fig. 4b) and CD4^+^ (not shown), but only a few of them were CD8β^+^ (Fig. 4b). The FCM analysis of the spleen cells of the same *rats* showed that TCRαβ^+^ and CD4^+^ donor T-cells were mostly EdU^−^ on day 1 but that many of them became EdU^+^ on day 2 (Fig. 4c, d). In contrast, CD8β^+^ donor T-cells were very few in number and rarely became EdU^+^ on day 2 (Fig. 4c, d), confirming the immunohistological finding (Fig. 4b).

### Analysis of cellular interactions between two different cell types

For analysis of cellular interactions in vivo, direct observation of different types of cells being activated through the interactions is crucial. We previously reported that early cluster formation of donor T-cells with host DCs and the proliferative response of these T-cells within the cluster represent the direct pathway of allosensitization in the allograft response (Saiki et al. 2001; Ueta et al. 2008). To reveal either two different surface markers of cycling S-phase cells or host DCs in the cluster, four-color immunofluorescence staining of the same *rat* spleens on day 2 was performed.

**Fig. 5:**
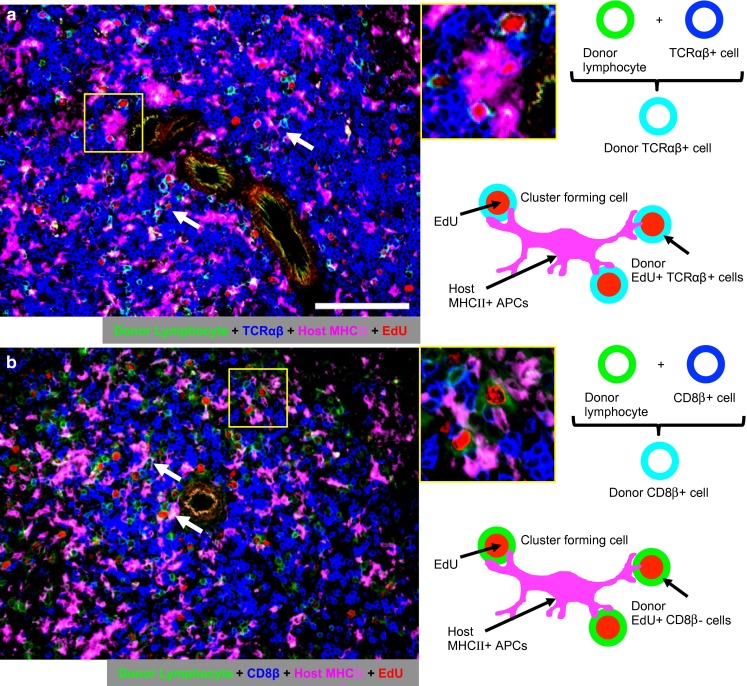
Phenotype of proliferating donor lymphocytes that cluster with host MHCII^+^ putative DCs (APCs). Four-color immunofluorescence staining of the spleen of day 2 after GvHR induction for TCRαβ or CD8β (Alexa-647-conjugated mAb, *blue*), donor lymphocytes (Alexa-488-conjugated mAb, *green*), host MHCII (indirect staining with Alexa-350-conjugated anti-mouse IgG, *magenta*), and EdU (Alexa-594-conjugated azide, *red*). Pseudocolors were assigned using AxioVision software. *Scale bar* 100 μm. Merged images are explained schematically in the right side of the panels. *Note* proliferation of TCRαβ^+^ (**a** *light blue* cell with *red* nucleus, *inset* and *white arrows*) but not CD8β^+^ donor T-cells (**b** *green* cell with *red* nucleus is CD8β^−^EdU^+^, *inset* and *white arrows*) that cluster with host MHCII^+^ cells

**Fig. 6:**
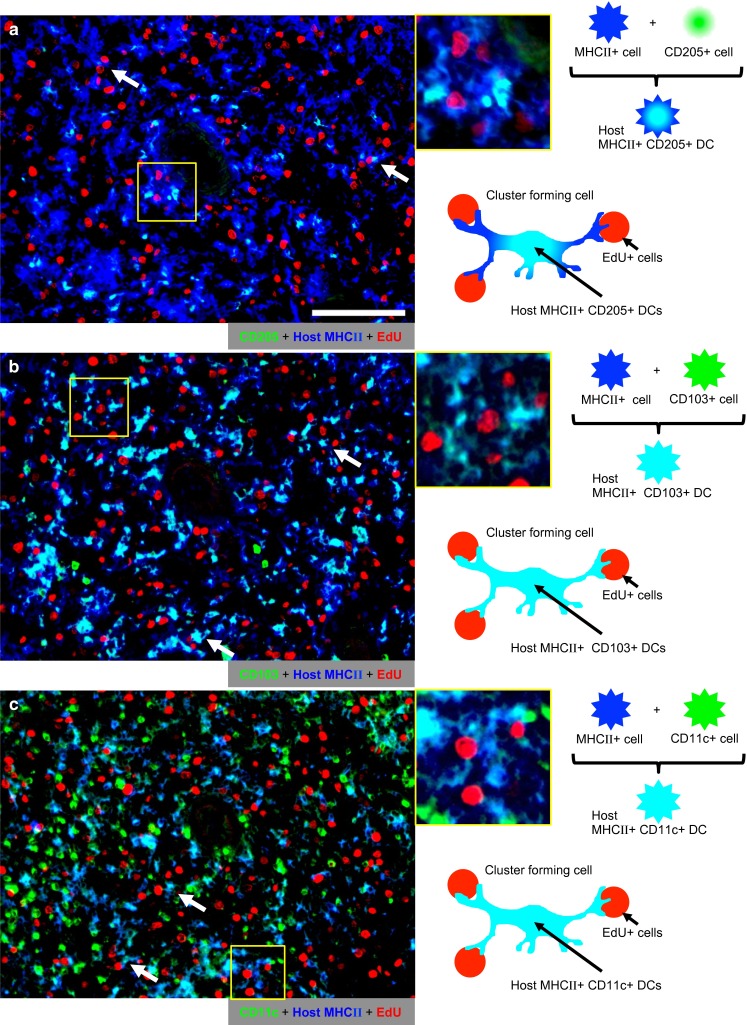
Phenotype of host MHCII^+^ putative DCs that cluster with proliferating cells. Three-color immunofluorescence staining of the spleen of day 2 after GvHR induction for host MHCII (Alexa-647-conjugated mAb, *blue*), CD205, CD103, or CD11c (indirect staining with Alexa-594-conjugated anti-mouse IgG, *green*), and EdU (Alexa-488-conjugated azide, *red*). Pseudocolors were assigned using AxioVision software. *Scale bar* 100 μm. Merged images are explained schematically in the *right side* of the panels. *Note* host MHCII^+^ cells that cluster with proliferating cells (*red* nuclei) are either CD205^+^ (**a**), CD103^+^ (**b**), or CD11c^+^ (**c**) (*light blue*, *inset* and *white arrows*)

**Fig. 7:**
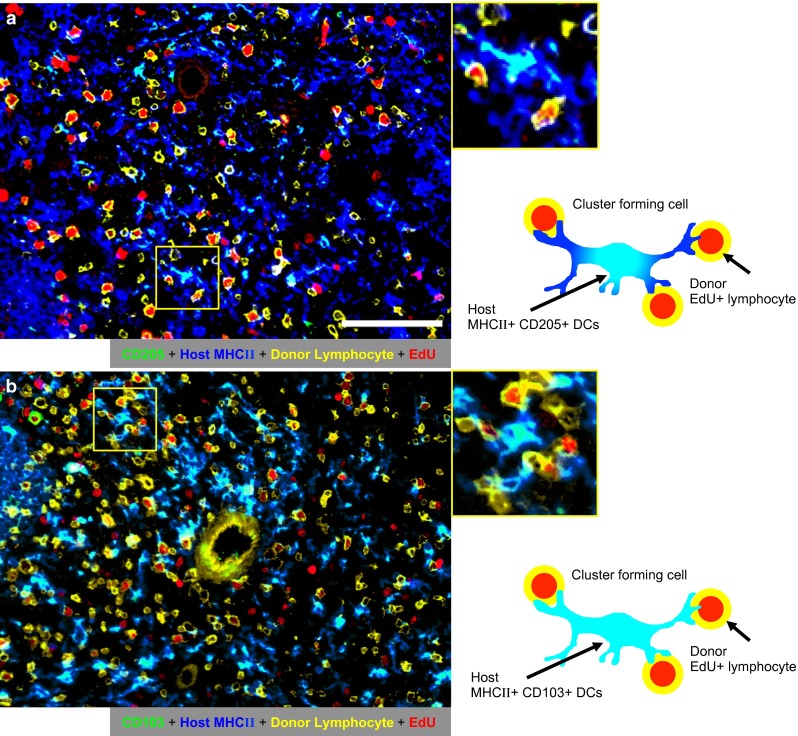
Cluster formation of host DCs with EdU^+^ proliferating donor lymphocytes. Four-color immunofluorescence staining of the spleen of day 2 after GvHR induction for host MHCII (Alexa-647-conjugated mAb, *blue*), CD205 or CD103 (Alexa-594-conjugated mAb, *green*), donor lymphocytes (indirect staining with Alexa-350-conjugated anti-mouse IgG, *yellow*) and EdU (Alexa-488-conjugated azide, *red*). Pseudocolors were assigned using AxioVision software. *Scale bar* 100 μm. Merged images are explained schematically in the right side of the panels. Note host MHCII^+^ cells that cluster with donor proliferating cells (*yellow* cells with *red* nuclei) possess DC markers, CD205^+^ (**a**) and CD103^+^ (**b**) (*light blue*, *white arrow*). Central *yellow ring* in **b** is the central artery, which emits nonspecific fluorescence

Concerning cluster-forming donor lymphocytes, many EdU^+^RT7^b+^TCRαβ^+^ cycling S-phase donor T-cells were seen to form clusters with host class II major histocompatibility complex antigen-positive (MHCII^+^) putative DCs in the PALS (Fig. 5a). In contrast, EdU^+^CD8β^+^ donor T-cells were few and did not form clusters with host putative DCs (Fig. 5b). As for the cluster-forming host MHCII^+^ cells with EdU^+^ cells, some of them possessed DC markers, such as CD205 (Park et al. 2012), CD103, or CD11c (Fig. 6), indicating that these cells were at least partly host DCs. Concerning the cluster-forming host DCs with EdU^+^ donor lymphocytes, cluster formation between host MHCII^+^CD205^+^ or CD103^+^ DCs and EdU^+^RT7^b+^ donor lymphocytes could be demonstrated (Fig. 7).

The results show that EdU staining enables analysis of cellular interactions in situ by simultaneous visualization of different surface markers of cycling S-phase cells or stromal cells.

## Discussion

In this study, by taking advantage of the unique characteristics of EdU, we have developed a new method that allows examination of immune responses with lymphocyte proliferation both functionally and morphologically in situ as well as in vitro. The four-color immunofluorescence staining using EdU for immunohistology or three-color for FCM and the simultaneous application of both staining approaches for one target tissue have not been reported so far. EdU staining correlated well with BrdU staining of spleen sections activated by the CD28SA stimulation (Supplementary Fig. 1). This outcome confirms previous indications that EdU can be used for immunohistology of cycling S-phase cells as a thymidine analogue (Salic and Mitchison 2008). The presence of some BrdU^high^EdU^low^ or BrdU^low^EdU^high^ cells might be due to the competitive uptake of both nucleosides by a single cell.

### Simultaneous detection in situ of two different markers of cycling S-phase cells

Because of the small size of the detection reagent and elimination of DNA denaturation steps, EdU staining allows the multicolor immunofluorescence of at least four colors including two different markers on a single-cell surface, which has been impossible by the standard BrdU method. In this way, any cells of specific phenotypes can be identified in situ, e.g., T-cells (Fig. 1), Tregs (Fig. 3), donor T-cells (Fig. 4b), and host DCs (Figs. 6, 7) and their functional molecules can be further studied, e.g., expression of CD25 (Fig. 3) and CD103 (Figs. 6, 7). Because these markers are mostly labile surface CD antigens, our method using fresh cryosections can provide a superb staining result for these antigens compared to the prefixation method used in the neuroscience field (Guo et al. 2009). Furthermore, some nuclear transcription factors can be simultaneously detected, e.g., Foxp3 in CD4^+^CD25^high^EdU^+^ cells (Fig. 3). These results show that this method enables functional time-kinetic analysis of immune responses of a certain cell type in a distinct domain of the lymphoid organs. An example is the Treg proliferative response occurring in the splenic PALS at day 3 after CD28SA stimulation (Figs. 2, 3).

### In situ analysis of cellular interactions between two cell types

Concerning the cluster formation between proliferating T-cells and DCs, the standard three-color immunoenzyme staining using BrdU could at best show only clusters of BrdU^+^ cells and host MHCII^+^ putative DCs (Saiki et al. 2001; Ueta et al. 2008). In the GvHR study, we could identify cluster formation between EdU^+^CD4^+^ donor T-cells and host MHCII^+^ putative DCs (Fig. 5), and the latter were further confirmed as host DCs, being either CD103^+^, CD205^+^ (Figs. 6, 7), or CD11c^+^ (Fig. 6). Therefore, the present method could allow analysis of cellular interactions in situ more precisely by the simultaneous visualization of different specific markers or functional molecules of cycling S-phase cells or stromal cells.

This cluster represents a site of antigen presentation by DCs to T-cells and proliferation and differentiation of activated T-cells (Saiki et al. 2001; Ueta et al. 2008), making it one of the most crucial structures when the afferent limb of the immune response occurs. Therefore, we propose that EdU staining can provide an exclusive method for clarifying essential cellular interactions in the immune response in vivo.

### Parallel analysis of FCM and immunohistology

Flow cytometry (FCM) analysis indicated that cycling S-phase cells could be detected by EdU staining at a higher signal-to-noise ratio due to click chemistry (Salic and Mitchison 2008) than ordinary BrdU staining that requires an antigen–antibody reaction. EdU staining also has enabled the parallel examination of the proliferative response of activated cells both by immunohistology of tissue sections and by FCM of cells derived from the same lymphoid organs of one animal. Thus, the immunoproliferative response was examined qualitatively by immunohistology of tissue sections, which then could be quantitated by FCM.

In this way, we could demonstrate not only the dose response of Tregs to CD28SA (Figs. 1, 2), but also the time kinetics and quantification of donor T-cell migration and proliferation in the GvHR (Fig. 4). In addition, some nuclear transcription factors detected by immunohistology were also detected by FCM and some proliferating cell subsets could be further isolated and used for RT-PCR analysis. Accordingly, this method can become a powerful tool for the objective and precise analysis of cellular interactions during the immune response in situ.

### Recommended protocols

**Fig. 8:**
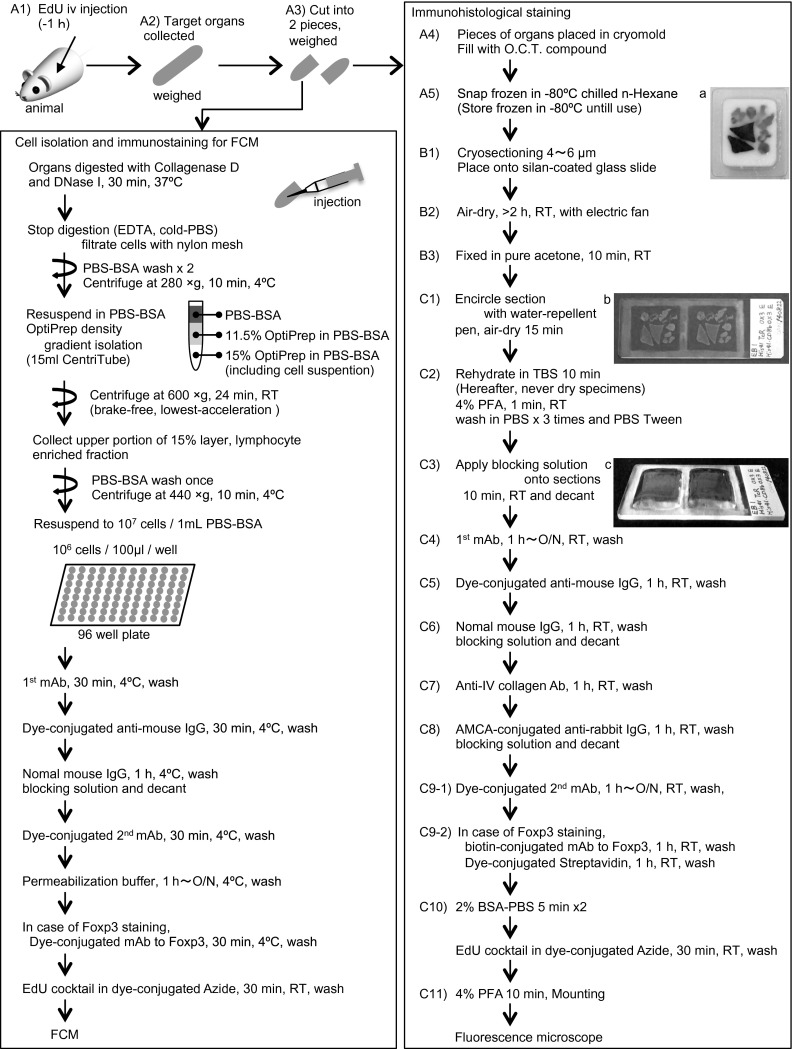
A flow diagram of sequential steps for the present method. *A1*–*A5*, *B1*–*B3*, and *C1*–*C11* correspond to the numbers in detailed working protocols described in supplemental materials

A flow diagram of sequential steps for the present method is illustrated in Fig. 8. Detailed working protocols with practical notes are described in supplemental online materials. To decrease the immunostaining steps, two mAbs of different species can be mixed and incubated simultaneously and then detected by a cocktail of secondary conjugates. Also, two different mAbs directly conjugated with different fluorochromes can be mixed.

## Conclusion

We have demonstrated a newly developed method that enables the functional time-kinetic analysis of immunoproliferative responses in vivo, including activation, proliferation, and cellular interactions in a distinct domain of the lymphoid organs, which are quantitatively confirmed by FCM. RT-PCR of proliferating cells can be analyzed further. This method is as easy and reproducible as standard immunofluorescence methods and would be applicable not only for the immune response but also for other studies examining cell and tissue growth, such as hematopoiesis and organogenesis.

## Electronic supplementary material

Supplementary material 1 (DOCX 98 kb)
