# Supplementary material for: A novel multicolor immunostaining method using ethynyl deoxyuridine for analysis of in situ immunoproliferative response
Source: Histochem Cell Biol. 2015 May 15;144(3):195–208. doi: 10.1007/s00418-015-1329-z (PMC4534512; doi:10.1007/s00418-015-1329-z)
Supplement: Supplementary file 1 — Supplementary material 1 (DOCX 98 kb) [file 418_2015_1329_MOESM1_ESM.docx]

**Supplemental online materials**

**Detailed working protocols with practical notes**

A. Tissue sampling

1. Inject EdU (5 mg/200 g body weight) intravenously and 1 h later, euthanize the animal by exsanguination under isoflurane anesthesia.

2. Excise target organs such as the spleen and lymph nodes and remove surrounding fat tissues as much as possible because fat does not freeze at ~ -20°C, the temperature for the cryosectioning, which results in difficulty in sectioning. Never pinch the target tissues with forceps, which causes destruction of tissues and results in poor morphology.

3. Weigh the organ and cut it in half. Weigh one piece to be used for FCM, by which total cell number and absolute number of the examined cell subsets can be estimated.

4. Trim tissue blocks of the organ suitable for sectioning and place into cryomolds filled with O.C.T. compound (Sakura Finetek, Tokyo, Japan). Different tissues can be grouped in one mold so that several tissues can be examined at once (Fig. 8a). These frozen blocks can be preserved in an airtight box for up to 5 years at -80°C.

5. Freeze tissues quickly in n-Hexane (Wako Chemicals, Osaka, Japan) chilled in a -80°C freezer. Alternatively liquid nitrogen or dry ice can be used for freezing.

B. Cryosectioning

1. Prepare cryosections at a thickness of 4–6 µm and place them on silane-coated slides (DAKO, Vienna, Austria) to prevent detachment. Keep the temperature of the cryostat chamber at -18°C, which is ideal for sectioning of lymphoid and many other tissues.

2. Air-dry specimens on slides for 2 h at RT with a humidity of <60%. For labile antigens, the shorter the drying time, the more intense the staining will be.

3. Fix slides in pure acetone (Wako) for 10 min at RT and air-dry for 5 min. Specimens can be preserved in an airtight box up to 1 y at -20°C or up to several years at -80°C.

C. Four-color immunofluorescence staining

1. For freeze-preserved slides, thaw the airtight box containing slides for 30 min at RT before opening. Encircle sections on a slide with a liquid-repellent slide marker pen (Daido Sangyo, Tokyo, Japan) just outside of the O.C.T. film margin and air-dry for 15 min (Fig. 8b). Drawing onto the O.C.T. film will detach the marking because O.C.T. is water-soluble. This step is to prevent spread of antibody solution over the slide and make wiping off of liquids around the sections unnecessary.

2. Rehydrate sections in Tris-buffered saline for 10 min and fix in 4% PFA for 1 min at RT, then wash in PBS three times. This fixation step hardens the sections and prevents their destruction during the staining procedure with little suppression of immunostaining for CD antigens. Perform each step in a Coplin jar. For the PFA solution, dissolve 20 g PFA powder (Merck, Darmstadt, Germany; 4005) in 400 mL distilled water with 200 µL of 1 N NaOH by heating and stirring (60–70°C). After cooling, add distilled water to make a final 500 mL solution and filter. The solution should be stored at 4°C and can be used for one month.

3. Rinse sections in PBS with 0.2% Tween 20 (PBS-Tween, Nakalai Tesque, Kyoto, Japan), then apply a blocking solution (Block Ace; Dainippon Seiyaku, Tokyo, Japan) onto sections and decant after 10 min. Before applying antibodies or any reagents, rinse sections in PBS-Tween each time. This step decreases the surface tension and reagents can easily spread over the slide surface.

4. Incubate sections with a first mouse mAb O/N at RT (Fig. 8c) in a moist chamber and wash three times in PBS. Perform every incubation in a moisturized chamber with slides horizontally placed in the dark. Never allow sections to dry out throughout the procedure, which will result in no staining. For washing, pour PBS in a Coplin jar containing slides, leave 2 min, and then decant. Repeat this three times. Two minutes each is enough for washing antibodies or fixatives.

5. Incubate sections with a fluorescent dye–conjugated secondary-mouse Ig antibody in PBS-BSA with 1% normal rat serum (blocking host-specific interactions) and incubate for 40–60 min at RT. Wash three times in PBS and rinse in PBS-Tween.

6. Incubate sections with normal mouse IgG (20 µg/mL) for blocking secondary antibody non-binding sites for 1 h at RT and apply a blocking solution and decant after 10 min.

7. Incubate sections with rabbit anti-type IV collagen antibody to outline tissue frameworks for 1 h at RT and wash.

8. Incubate sections with aminomethylcoumarine–conjugated anti-rabbit IgG antibody for 1 h at RT and wash. Apply a blocking solution and decant after 10 min.

9-1. Incubate sections with a second mAb directly conjugated with a fluorescent dye for 1 h at RT and wash.

9-2. In case of Foxp3 staining, incubate sections with biotin-conjugated mAb to mouse/rat Foxp3 for 1 h at RT and wash. Then, incubate sections with Alexa-488–conjugated streptavidin for 30 min at RT.

10. Apply PBS with 2% BSA for 5 min twice. Then, EdU is stained for imaging by incubating sections horizontally with ~100 µL per section of the Click-iT kit solution.

11. The sections are fixed in 4% PFA for 10 min to prevent destruction during microscopic observation, washed, and cover glass mounted with Fluorescent Mounting Media (KPL, Gaithesburg, MD).

Notes

In principle, any combination of antibodies and secondary conjugates is applicable provided the species of mAbs in use are different each other as employed in the standard FCM analysis. In case two mouse mAbs are necessary reagents, the first mAb is detected by the indirect immunofluorescence staining followed by blocking of the antigen-binding site of secondary antibody with normal mouse Ig. Then the second mouse mAb directly conjugated with a fluorochrome is reacted. Alternatively, two mAbs of the same species can be used where one is biotin-conjugated followed by a fluorochrome-conjugated streptavidin and another is directly conjugated with a different fluorochrome.

**Supplementary Figure Legends**

**Supplementary Fig. 1** EdU staining correlates well with BrdU staining in immunohistology. Triple immunofluorescent staining for BrdU (Alexa-647–conjugated mAb *red*), EdU (Alexa-594–conjugated azide, *green*), type IV collagen (indirect staining with AMCA–conjugated anti-rabbit IgG, *grey*) and a merged image. Splenic PALS on day 3 after CD28SA injection (0.5 mg/*rat*). Note superimposition of EdU^+^ nuclei on almost all BrdU^+^ nuclei. *Scale bar* 100 μm.
